# Putting the Bumps in the Rocky Road: Optimizing the Pathway to Excellence

**DOI:** 10.3389/fpsyg.2016.01482

**Published:** 2016-09-28

**Authors:** David J. Collins, Aine Macnamara, Neil McCarthy

**Affiliations:** ^1^Institute of Coaching and Performance, University of Central Lancashire, PrestonUK; ^2^Grey Matters Performance Ltd, Stratford-upon-AvonUK; ^3^Gloucester Rugby Academy, GloucesterUK

**Keywords:** challenge, periodization, psycho-behavioral skills, talent development environment, psychologically robust

## Abstract

There seems to be general agreement on the importance of challenge for effective development on the athlete pathway. What seems less coherent, however, are ideas on how much, when and how this challenge should be used. Reflecting our own experience as applied practitioners and our ongoing research, we offer a perspective on this work from a practitioner stance. The literature suggests that differences between levels of adult achievement relate more to what performers bring to the challenges than what they experience. Therefore, it is essential that young athletes have the opportunity to develop psycho-behavioral and coping skills, and have adequate social support, to ensure that adversity is interpreted as a positive growth experience. A periodized and progressive set of challenge, preceded with specific skill development, would seem to offer the best pathway to success. The importance of preparing athletes for challenges, supporting them through the experience, and then encouraging positive evaluation and reflection is key to successful outcome. Finally, we offer some suggestions, structures and systems, which can be used to support the skill-based approach promoted.

## Introduction

There is increasing support for the importance of challenge in the development of high potential young performers (e.g., Collins et al., 2016). What is less apparent, however, but essential from the practitioner perspective, is how the Talent Development Environment (TDE) can be designed and deployed to maximize the benefit of such challenge; in short, some guidelines on how challenging bumps in the road can best be placed and/or exploited.

## Theories of Development: The Current State of Play

As a starting point, we would suggest that the use of challenge in TDEs should be purposeful and carefully considered, rather than based on *post hoc* correlations and reports. Accordingly, and to better situate the skills-based approach espoused in this paper, we first offer a brief review of some of the standpoints currently apparent. For the present purpose, we will group the in vogue approaches into three broad groups: life experience, attitude and skills. While not probably reflective of the original authors’ epistemological stance, our structure holds face/construct validity and facilitates a critical appraisal of each against the needs of the sports practitioner.

### Life Experiences

The life experience stance sees high achievement as due to perhaps serendipitous experiences of developing performers. The current resilience literature of Fletcher and Sarkar (2012) and Sarkar and Fletcher (2014) is a good example of this. The authors used primary data plus auto-biographies and biographies of high level performers to demonstrate the positive results of challenge (e.g., Howells and Fletcher, 2015). As per the title on one of their papers, these benefits were presented as “What doesn’t kill me makes me stronger” (cf. Sarkar et al., 2015). Our concern here is that it might more often kill you instead! In short, it is important to understand how much stress, of what kind, when and how dealt with will generate optimum benefit. Furthermore, can the athlete be prepared in advance to benefit, or are the skills an accident of birth and upbringing? Notably, the relationship between stress and consequent resilience seems to be curvilinear, with optimum growth resulting from moderate levels (too much or too little and benefits are less apparent – Seery, 2011). Furthermore, effects can often be due to cumulative rather than just single occurrence, acute stress (Seery et al., 2010). Of course, moderate is a relative and often perceptually mediated term, so it would seem that a trait tendency (such as proactive coping which can be taught – e.g., Greenglass and Fiksebaum, 2009) or learnt skill (Rosenbaum, 1983) would be crucial in determining the outcome. Finally, the existence of challenge is not, in and of itself, a consistent trigger for positive growth. Even if initiated, the process of development is likely to be complex and long term; it is certainly not a static or short term outcome (cf. Tedeschi and Calhoun, 2004).

Offering another perspective on the life experience view, and based on interviews with 16 multiple medalists contrasted with 16 Olympian non-medalists, the UK Sport sponsored Great British Medalists study of Rees et al. (2013) proposes the early juxtaposition of sporting success and impactful trauma as a universally common developmental experience and discriminating factor between these successful and less successful peers. As the report summarizes;

although a happy childhood may be a good thing, the overcoming of obstacles and difficulties (critical events) may underpin the mental toughness, resilience, and deep-seated need to achieve, which serial gold medalists may possess. (Key Point 3).

Our experiences made us very surprised at this statement, representative as far as we understand their meaning, of every serial medalist they studied. As an interesting contrast, a parallel study comparing triads of super-champs (multiple medalists or multi-capped, team sport athletes), champions (single medal/low number of caps) and almosts (no senior medals or caps – Collins et al., 2016) found little evidence for this effect. Indeed, almosts reported more trauma than super-champs, although even then only as a moderate percentage of the whole sample. Concerningly, from an applied perspective, descriptive work such as this, and the purposeful biographical reviews completed by Howells and Fletcher (2015), may offer comparatively little to the field, representing a focus akin to the ‘great person’ work which characterized the early days of leadership research (Bass, 1990) in describing rather than driving the development of such individuals.

### Attitudes

The second approach centers on the role of attitudes in mediating individuals’ responses to challenge and adversity on the pathway to excellence. Considerable attention has been paid to constructs such as grit (Duckworth et al., 2007) and they are increasingly used ‘buzz words’ in talent development. Through such constructs, an emphasis is placed on self-discipline, will power, persistence and the ability to defer gratification as key attributes for young athletes. Simply, performers high in these constructs seem more likely to get to the top and achieve more when they get there. For example, grittier individuals, defined as those with perseverance and passion for long-term goals, seem to achieve more than their less gritty peers across a variety of domains (e.g., Eskreis-Winkler et al., 2014). However, what is less clear from a practitioner perspective are guidelines about the teaching and development of these attitudes as preparation for challenge. Simply, despite evidence that these constructs are important for overcoming developmental challenge, there is little consideration of how they are developed within the TDE.

Beyond the lack of understanding of the mechanisms underpinning these constructs, it is also important to consider the downside to grit; persistence may be counterproductive, termed non-productive persistence (McFarlin et al., 1984), especially when inappropriate. In fact, more recent reviews suggest the effect is largely due to persistence rather than grit as a distinct construct (Credé et al., in press). In the present context, gritty individuals, especially those on a very competitive developmental pathway, may show persistence at a task, or in overcoming challenge, resulting in unremitting failure or inefficient success that could have been surpassed by alternative courses of action (McFarlin et al., 1984). As such, there seems to be some benefits to knowing when not to persist at a task from both an outcome and an individual perspective (i.e., well-being; Miller and Wrosch, 2007; Hill et al., 2015).

### Skills

The third category of work is skills focused and, as such, offers more explicit guidance to the practitioner on what to do. As an example, the work of Toering et al. (2009; see also Duckworth et al., 2010) which shows that self-regulatory skills distinguish between elite and sub-elite academy footballers. A subsequent, more detailed study suggested that these differences operate, at the least, through the generation of better practice behaviors (Toering et al., 2011). This suggests that developing such skills in young performers offers them the equipment to make the most of their pathway experiences.

A similar picture is provided by the work of MacNamara et al. (2010a,b), albeit that their Psychological Characteristics of Developing Excellence (PCDEs) offers a broader range of structured skills. Once again, however, the crucial point is that skills are taught, tested, refined and redeployed as the athlete progresses along the pathway, using both naturally occurring (e.g., MacNamara and Collins, 2010) and constructed challenges (cf. Collins et al., 2012), rather than development being ‘left to chance’ if and when opportunities occur, or being seen as due to existing personality traits.

Of course, in summarizing this brief overview, we should acknowledge the possibility that all three of the approaches are saying the same thing. In short, that whether the skills are acquired through life experience, emerge as an attitude or are formally taught, it is the skills, and the ability/willingness to use them, that makes the difference. We will leave more detailed consideration of this to another paper. For the moment, however, we would reiterate that the first two routes are, at best, leaving the development of these key causative factors to chance. So, with this possibility in mind, what evidence exists for the skills based approach?

#### Evidence in Support of the Skills-Based Approach

There is emerging evidence that the skills described in the previous section can be taught, with consequential impact on performer’s ability to cope with developmental challenges. Given the positive implications for the application of these skills, building these into TDEs should be a feature of effective programs. There is evidence for the impact of such skill-based developmental programs. For example, the Developing the Potential of Young People in Sport (DPYPS – Collins et al., 2010) pilot program, which used physical challenge and taught PCDEs in an integrated approach to young school children. PCDEs were formally taught, encouraged, modeled and refined, then transferred and tested using a variety of means in both curricular physical education and extra-curricular sport. Results showed that young participants were able to apply PCDEs to a wide range of challenges, from both within and outside their sporting environment, which helped them maintain progress and development. Of course, these skills and psychological robustness may also be developed by virtue of the pathway. For example, McCarthy and Collins (2014) suggest that the attritional journey experienced by relatively younger athletes acts as a catalyst for the development and deployment of coping strategies, psychological resilience and “mental toughness.” Overcoming high levels of challenge due to the physical and cognitive loads experienced by relatively young athletes may be actually beneficial for long-term development as it provides opportunities to develop, deploy and refine PCDEs required for long-term development.

There are also several strands of parallel research, which support the skills approach. For example, levels of hardiness (i.e., commitment, belief in control and enjoyment of challenge) discriminate successful athletes (Sheard and Golby, 2011), so it is likely that any intervention that builds these would support the pursuit of higher levels of achievement. The tendency for initial appraisal of stress as a challenge as opposed to threat is also concomitant (e.g., Kassam et al., 2009), or maybe even causative (cf. Greenglass and Fiksebaum, 2009) of positive outcome. Once again, the development of these attitudes through skills training, challenge, support and reassurance would seem desirable.

Of course, we should stress that no one answer can represent the total solution. Clearly, the provision of support to athletes is a subtle and complex issue; certainly not just a case of “teach them some skills and watch them grow!” Indeed, as Güllich and Emrich (2006) show in an important and rare large-scale quantitative survey, certain support structures and procedures may lose or even reverse effects over time. In similar fashion, there are a range of individual differences which mediate the impact of mental skills training on performance (Geukes et al., 2012; Roberts et al., 2013), and some subtle differences exist between facilitative and debilitative versions of the same construct (e.g., perfectionist strivings versus concerns, Stoeber, 2011). These complicating factors underpin our insistence on an *individualized* treatment to the development and deployment of skills.

In acknowledging these complexities, however, we still suggest that a concentration on the possession of, confidence in, and ability to deploy skills against increasingly varied types of challenge offers the best-applied focus for practitioners. Indeed, the post-traumatic stress literature implies the deployment of skills, many of which are taught by the therapist to the client, to facilitate the transformation of stress into growth. As Joseph et al. (2012, p. 319) state “affective–cognitive processing takes place via the cycle of event cognitions appraisal, emotional state, and coping as the person attempts to reconcile pre-trauma-related assumptions with the new trauma-related information.” Their model highlights the complexity of this process, stating that a variety of methods may work, or even be required, both between and within individual. In other words, the transformation from stress to growth is far from automatic, and seems dependent on the input of well-trained and well-informed practitioners.

## The Best Way forward? Periodized Challenge in a Kolb-Like Cycle

Reflecting and integrating the best elements of all these approaches, we propose a teaching, challenging, evaluating and refining cycle which matches the traditional Kolb Cycle (Kolb and Fry, 1975) in experiential education. Through this medium, young athletes experience a gradual development of skills, which are then tested against realistic (rather than contrived) challenges. After the challenge, coaches and other practitioners engage the athletes in review, developing their own capacity to evaluate and self-manage in tandem with structured feedback. Given the need for reflection and refinement, this approach is built around a periodized use of challenge, allowing sufficient time for athletes to learn from, develop and refine and, crucially, secure confidence in their capacity to use the skills. As such, it seems to us that periodization, a construct increasingly challenged in its home discipline of physiology (Kiely, 2010), can enjoy a new lease of life in a psychological application for athlete development.

It is important to note that this approach needs to be a lot more than merely the provision of mental skills or cozy chats about mindsets. Work must be one-on-one as much as possible, to help the individual to explore, discover and build confidence in the particular blend which works best for them in their particular environment. This, in turn, raises the need for regular and ongoing refinement or even revision as people grow and situations change.

### Building and Deploying the Speed-Bumps – How the Impact of Challenge Can Be Optimized

Reflecting the increasing support networks that characterize the TDE landscape, the task is one of pulling the various support disciplines and influences together in a coherent and coordinated manner to best support and provide suitable challenge and opportunity for an athlete. Given the complexities of both process and landscape, this becomes a challenge in itself for practitioners who seek to optimize and drive a genuinely individualized program. A key element of this process is the performance manager (PM)^1^, whose role is one of orchestration for the various support agents, as architect of the Individual Action or Development Plan (IAP/IDP) and, perhaps, as chair of the Case Study Reviews (CSRs) process in conjunction with the athlete. For younger athletes, the PM should also be responsible for managing parents and other influences in a manner that best supports continued adherence to the long-term objectives (Pankhurst et al., 2013). In a well-run system, these will be clearly defined in the IAP and managed closely through the CSR process. Care is needed to ensure genuine challenge and growth from this process; in recent times, over management of athlete journeys has been a strong but often detrimental focus for development systems, with eliminating non-sport-related challenges a priority (cf. Collins and MacNamara, 2012). With this caveat addressed, we suggest that a well-planned, well-managed, periodized and individualized challenge strategy has strong merit in the long-term development of more psychologically robust athletes.

When designing challenging periods, examples can be as simple as encouraging/holding back athletes to compete at levels above or below their current age grading, either through training or exposure in competitions. This can be achieved by drip feeding players into senior level competitions or training for short periods of time and/or with specific targets set. Even simply warming up at major senior events with senior athletes or participating in an element of their technical skillset at intervals independent of the competition event itself (penalty kicking at half time for example) can have a significant impact. It is also important to periodically test for progress by placing athletes in surroundings familiar to them (back into their own age grading, for example), having spent periods of time away under increased ‘challenge.’ This return can provide competition or training opportunities at a level whereby an athlete is freely able to express newfound experiences and confidence, whilst allowing a period of adaptation is essential to maximize opportunity for growth. Periodizing challenge in this manner can be positive both for the individual and the other squad members, potentially accelerating development. A well-structured plan can apply any number of periodized blocks throughout an athlete’s development journey.

Of course, challenge is and should not be without stress, so such mechanisms for building coherence across the support network are essential to maximize the type and nature of challenge that can be applied by design for optimal adaptation. A developmental plan of this nature should not be all about challenge, however. We would suggest this is best served through effective case conferencing of the athlete, inclusive of all support personnel, but in full acknowledgment of the competing demands they experience. In many cases these may be outside the control of the primary provider (e.g., a club v country scenario) but nonetheless are prominent and sometimes aside from the athlete’s sporting aspirations (school v club or country). What is essential as an outcome through the case conferencing process is a clearly defined plan that is inclusive, coherent and which gains general commitment. Well-intentioned support personnel can inadvertently undermine the whole process through not adhering to the agreed development plan and going off ‘task,’ especially during periods when the athlete is seeking homeostasis.

One other important consideration is the degree to which the stress can be seen as truly developmental. There are clear examples of excessive stress, bordering on or even exceeding the line of abuse, in youth sport settings (Stirling and Kerr, 2015). These authors also highlight the ways in which such stress can have long lasting negative effects, even though apparently innocuous at the time. We would certainly concur with the second point; surely also a particular consideration in the argument against transformative stressful life experiences. To reiterate, they might not make you stronger at all. We will examine exactly how much stress is good for you in a later paper. For the moment, however, we would repeat the need for careful performance management of the young athlete, with the system ensuring that tests of skills are carefully debriefed and next phase challenges primed.

Such protective mechanisms notwithstanding, all this suggests a cluttered and sometimes distracting atmosphere that can, in some instances derail athletes from their primary objectives. Whilst acknowledging the merits of being intrinsically motivated, even the most motivated individual requires guidance and advice when attempting to negotiate a way through the clutter around the development journey. Accordingly, management needs to drive, monitor and maintain a coherent pathway across the different stages encountered on the way up (Webb et al., 2016).

So, as a simple example, consider this micro intervention; part of our work with players at a premiership football academy. The coach ‘surprised’ his players (who have just joined him at the start of a new season) with an aerobic fitness bleep test. Used, of course, to provide a fitness baseline but, more importantly, followed through and exploited via a variety of group and individual debriefs. Concepts such as impression management, plus PCDEs such as commitment, imagery, preparation and goal setting, were explored and action plans made with individuals for future unannounced tests. Our point here is that a fairly normal challenge can be exploited to good effect, by coach and psychologist working in tandem. Follow ups through player’s social media suggested that the ideas had stuck, whilst their continuing use of the terms in normal social conversation and applications to football training show good transfer. Repeat this type of process in a cyclic fashion and a positive spiral of skills and attitude can be generated.

## Conclusion – Where Next (Or Not)?

We hope to have made a case for a skills-based approach to optimizing challenge in TDEs. There is already a strong base for the approach and ongoing investigations seem only to reinforce its practicality and impact. Acknowledging the limitations of the vast majority of studies cited in this paper (i.e., retrospective designs, recall bias) while also recognizing the difficulty of control when working in applied settings, perhaps the best approach for future research in this area is a triangulation of longitudinal tracking, plus theoretical grounding (evidence that this is a logical and effective approach) plus coach perceptions (given that their experiences run across individuals) plus a focus on success stories and failures. Essential for the skills approach are prospective and longitudinal studies which test the success and failure of athletes against the possession and emergence of improved skill sets. Use of validated instruments such as the Psychological Characteristics of Developing Excellence Questionnaire (PCDEQ –MacNamara and Collins, 2012) should demonstrate greater success for athletes in possession of higher scores. Furthermore, and increasing the opportunities to test for causation, following the ideas described in this paper should generate higher scores and greater success. These ideas feature in where we are currently going with our own research.

The inherent bias of asking someone about a pathway through which s/he has succeeded (e.g., MacNamara et al., 2010a,b; Rees et al., 2013; Howells and Fletcher, 2015; cf. Bailey and Collins, 2013) is a particular weakness which needs addressing. The recounted experience has, of course, been effective for that individual and so, will be supported. Unfortunately, however, in the absence of other experiences, the participant cannot really be expected to comment critically on the strengths and weaknesses of the approach they experienced; only on how it felt for them. As such, even longitudinal tracking research in this environment must be read with caution and findings should not provide a conclusive case for or against certain approaches.

We must also raise questions as to the inherent bias which, we believe, may be almost inevitable within biographical accounts. It seems logical that the desire to sell the book and to present the subject in the best light may inevitably lead to, at best, presentational bias and, worse still, hyperbole about life experiences and their impact. Thus, whilst casting no doubts on the interpretation made by Howells and Fletcher (2015) in their increasing use of such sources, we must question whether this is a genuine and effective means of developing approaches for the future. At the very least, such retrospective reports are surely both contextually dated and individual-specific! In short, and at the very least, some careful consideration is needed before they are uniformly applied

In concluding, we should also point out the urgent need for such work, as the ideas of talent and trauma gain leverage in the popular psyche. In fact, we would suggest that this is already taking place, with the popular press increasingly stressing how important ‘experts’ see early trauma in the development of young athletes (e.g., Daily Mail, 2016). The traction of the Great British Medalists study (Rees et al., 2016) is also a concern to us. Certainly a prestigious sample, but a single qualitative retrospective with 32 Olympic participants surely needs greater corroboration before it is used to drive policy and practice. These concerns are particularly pertinent when the results are uncritically extended to team sports without further work. In short, there is a potential for the trauma ideas to lead practitioners (and researchers) astray, even generating the kinds of concerning abuse highlighted by Stirling and Kerr (2015). Further investigation and critical debate is needed. We hope that this paper stimulates such conversation. In the meantime, we commend the ideas in this paper to scientist-practitioners everywhere.

## Author Contributions

All authors listed, have made substantial, direct and intellectual contribution to the work, and approved it for publication.

## Conflict of Interest Statement

The authors declare that the research was conducted in the absence of any commercial or financial relationships that could be construed as a potential conflict of interest.

## Footnotes

1 We use acronyms in common usage within the TDEs in which we work. Others are, of course possible, although meanings and usage may vary across systems.
